# Ultrasonic Welding of Novel Carbon/Elium^®^ Thermoplastic Composites with Flat and Integrated Energy Directors: Lap Shear Characterisation and Fractographic Investigation

**DOI:** 10.3390/ma13071634

**Published:** 2020-04-01

**Authors:** Somen K. Bhudolia, Goram Gohel, Jayaram Kantipudi, Kah Fai Leong, Robert J. Barsotti

**Affiliations:** 1 School of Mechanical and Aerospace Engineering, Nanyang Technological University, 50, Nanyang Avenue, Singapore 639798, Singapore; goram001@e.ntu.edu.sg (G.G.); kant0004@e.ntu.edu.sg (J.K.); MKFLEONG@ntu.edu.sg (K.F.L.); 2 Institute for Sports Research, Nanyang Technological University, 50, Nanyang Avenue, Singapore 639798, Singapore; 3 Arkema Pte Ltd., Singapore 117528, Singapore; robert.barsotti@arkema.com

**Keywords:** polymer-matrix composites (PMCs), thermoplastic resin, ultrasonic, joints/joining

## Abstract

The current research work presents a first attempt to investigate the welding attributes of Elium^®^ thermoplastic resin and the fusion bonding using ultrafast ultrasonic welding technique. The integrated energy director (ED) polymer-matrix composites (PMCs) panel manufacturing was carried out using the Resin Transfer Moulding (RTM) technique and the scheme is deduced to manufacture a bubble-free panel. Integrated ED configurations and flat specimens with Elium^®^ film of different thickness at the interface were investigated for ultrasonic welding optimization. Optimised weld time for integrated ED and flat Elium^®^ panels with film (0.5 mm thick) configuration was found to be 1 s and 5.5 s, respectively. The ED integrated configuration showed the best welding results with a lap shear strength of 18.68 MPa. The morphological assessment has shown significant plastic deformation of Elium^®^ resin and the shear cusps formation, which enhances the welding strength. This research has the potential to open up an excellent and automated way of joining Elium^®^ composite parts in automotive, wind turbines, sports, and many other industrial applications.

## 1. Introduction 

Thermoplastic (TP) composites are mostly preferred in different manufacturing industries, due to their excellent damping, impact, fracture toughness, recyclability properties, and its ability to be fused or welded to itself or with other materials [1,2]. Thermoplastic resin has the ability to soften once heated above the defined temperature range and retain its properties once it is cooled down. Hence, the TP composite is an attractive candidate for the welding of two similar TP composite materials and also with dissimilar materials, like thermoset (TS) composites and metals. There is a growing call from the wide spectrum of industries (aerospace, automotive, sports, and many more) to eradicate the classical ways of joining the polymer composite parts, namely mechanical fastening and the use of control adhesives. The major drawback of using the former is that, due to the holes and cutouts in the composites, they are susceptible to high-stress concentration and it is also labour intensive, whilst the latter requires an incredibly longer curing time as well as the longer surface preparation [3,4]. The welding attributes of thermoplastics aids the cost-effectiveness of the composite part to be manufactured in an industrial environment from forming until the finishing steps [1,5,6,7]. The most feasible welding techniques that are available for fusion bonding of thermoplastic composites are resistance [8,9,10], induction [9,11,12,13,14,15,16], and ultrasonic welding [7,17,18,19,20,21,22,23,24,25,26,27,28,29,30,31,32,33,34,35]. They behave differently in a way the heat is generated at the welding interface. 

Ultrasonic welding is an ultrafast joining process for thermoplastic composites. The working principle of ultrasonic welding involves the use of high-frequency (commonly 20 kHz) mechanical vibrations that are transmitted through thermoplastic parts to generate a frictional heat build-up at an interface, which will help the thermoplastic material to melt and flow and then forms the interfacial bond between them [36]. Ultrasonic welding possesses distinct advantages over other fusion bonding methodologies, such as the higher weld strength, it can be bonded in few seconds, and it is independent of the use of particular extra material at the interface, as required in electromagnetic welding [37]. 

Energy director (ED) is a protrusion of the polymer on the laminate surface, which required for obtaining a good bond as it concentrates ultrasonic energy at the interface. ED is an important physical parameter that improves the welding quality. Chuah, Y.K. et al. studied the effect of different parameters on the welding strength of acrylonitrile butadiene styrene (ABS) and polyethylene (PE) [38]. Three different energy directors were investigated—semi-circular, triangular, and rectangular—and the most efficient was found to be the semi-circular ED followed by rectangular and triangular configuration [38]. Villegas, I.F. et al. have studied the influence of several configurations of energy directors and investigated the effect of direction of the energy director with respect to the load direction [25]. They also studied the effect of the size and distribution of multiple energy directors. The results were examined by lap shear testing and no significant effect of the direction of the ED on the lap shear strength (LSS) value was noticed. Whereas, the laminate with multiple ED showed an increase in welding strength increase with the increase in the ED volume up to a certain threshold limit, and a further increase in the ED volume resulted in a reduction of 34% in the LSS value. Many researchers studied the effect of flat ED or flat film of different thickness [34,35,39,40] on the ultrasonic welding of thermoplastic composites. Goto, K. et al. investigated the shear and tensile strength of the ultrasonic welding of the CF/PA6 composite with flat ED (PA6, 0.3 mm film) and without ED at different weld energies [34]. Their study showed that the LSS1 value is around 77% higher with flat ED as compared to the adherends welded without ED, while LSS2 showed almost the same result. Palardy, G. et al. [40,41] studied the effect of flat energy directors while using the displacement control mode with different material systems. Palardy, G. et al. investigated the effect of the thickness of the flat ED of PEI, 0.06, 0.25, and 0.5 mm on the ultrasonic welding and studied the power curve [40]. It was deduced from the study welding with the thicker ED films 0.5 mm, they melt first, and then the substrate. While, in the case of 0.06 mm, the film and adherends melt simultaneously.

The ultrasonic welding time is one of the key parameters in the ultrasonic welding process [42]. Tao, W. et al. [35] investigated the effect of different welding time on welding strength of carbon fibre reinforced Polyether ether ketone (PEEK) composite with flat PEEK ED by keeping the other parameters constant. This study showed that the welding quality increases with the increase in time from 0.7 s to 0.8 s, but further at higher welding time (1.1 s) large cracks and voids are formed at the interface; 0.9 s was found to be the optimum time for good weld quality. Harras, B. et al. [18] showed that the optimum joint strength is more related to the total energy input parameter in the welding process than the weld time. Wang, K. et al. [32] showed that welding energy is one of the dominating parameters for obtaining good quality welding by using a two-level full factorial experiment. Liu, S.-J et al. [21,42] investigated the effect of different welding parameters, like weld time, weld pressure, the geometry of the ED, amplitude, hold time, and hold pressure on the weld quality while using the Taguchi method. The materials used for the study were polypropylene reinforced glass fiber composites and Nylon 6 reinforced glass fiber composites. The investigation showed that weld time, the amplitude of vibration, and ED geometry have significant effect on the weld quality. 

Recently Arkema developed a novel acrylic thermoplastic resin, Elium^®^ which is a first of its kind TP resin system to cure at room temperature and it possesses the same in-plane mechanical properties as compared to the high-performance epoxy resin. It can be manufactured while using liquid injection processes, like Resin transfer moulding and vacuum infusion processes, as it possesses a viscosity that can go as low as 50 cP. Already, significant research is reported in the literature investigating the impact [43,44,45,46,47], fracture toughness [48,49,50], vibration [51], flexure [52,53,54], tensile [54,55], and other attributes [56,57] of this novel resin system with different fibre reinforcements. Murray, R.E. et al. investigated the flexural characteristics of GF/Elium composites for tidal wind turbine blade applications [58]. Flexural stiffness for the epoxy spar cap was found to be 43 GPa as opposed to 40.4 GPa for thermoplastic Elium spar cap. A research study was carried out on the fusion bonding of the Elium^®^ composite, by Murray, R.E. et al. [59]. In this research, resistance welding using different heating elements and induction welding techniques were used to investigate the welding attributes of Glass fibre Elium^®^ composites for wind turbine blade applications. It was noticed from the study that there was a 30% improvement in lap shear strength for the welded samples as compared to the one bonded with adhesives. At 10 million cycles (defined stress for no failure), the fatigue limit for a fusion-welded sample was also found to be 5 MPa as compared to 3 MPa in the case of the adhesively bonded sample.

Recently, a study was carried out by the Bhudolia, S.K. et al. [60] on ultrasonic welding of the carbon/Elium^®^ composite using one configuration of integrated ED and its comparison with the conventional adhesive joint. The results showed a 23% higher lap shear for an ultrasonically welded specimen as compared to the adhesively bonded joints. Additionally, Bhudolia et al. analysed the fatigue response of the welded sample and compared the integrated ED and adhesively-bonded sample [61]. The result showed a higher fatigue life at a given load as compared to that of the adhesive joints.

It is of paramount importance to investigate the effect of flat energy directors on the welding, as using the energy directors is not a good solution, especially for the part with complex geometries. The current research presents the first attempt at manufacturing different configurations of integrated ED carbon/Elium^®^ laminates and comparing its welding properties to the welded configurations that are fused using a flat energy director. Additionally, a detailed investigation is carried out on understanding the effect of the different types of EDs on the welding attributes of the novel carbon Elium^®^ composite. Current research paves an excellent way of joining Elium^®^ composite parts. It could be significant for wind turbines, automotive, sporting, and other applications where the composite parts are currently joined using sophisticated control adhesives. 

## 2. Materials and Manufacturing 

### 2.1. Materials

For thermoplastic composite manufacturing, FOE sized 12K 2 × 2 twill weave dry carbon fibres that were supplied from CHOMARAT of 380 gsm [62] were used as a reinforcement and Elium^®^ 150 resin, provided by Arkema, France as a matrix material. Elium^®^ 150 is a liquid thermoplastic resin, a recent invention from Arkema is an acrylic-based resin and the first of its kind that can be cured at room temperature. It undergoes radical polymerization to form high molecular weight acrylic co-polymers with a benzoyl peroxide initiator at a mixture ratio of resin to hardener 100:3 [5,63]. Table 1 summarizes the important properties of the Elium^®^ 150 resin. Resin XB 6078, Huntsman was used as the binder material to bind the fibre preform in current research.

### 2.2. Manufacturing of Flat Laminates

**Figure 1:**
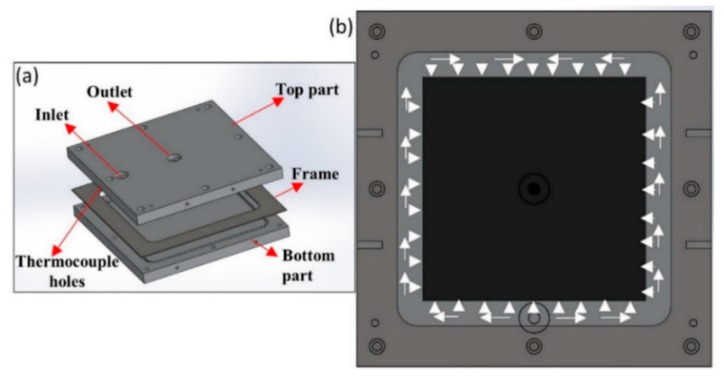
(**a**) Mold design for the flat composite laminate manufacturing (**b**) circumferential resin strategy.

Flat composite laminate was manufactured while using a three-part RTM mould i.e., top part, bottom part, and a frame of thickness 2 mm (refer Figure 1a). Before manufacturing the laminate, mould surface preparation was carried out to ease the demoulding once manufactured. Five layers of dry carbon fiber layers were cut and placed into the mould with a frame of 2 mm to achieve the required fiber volume fraction of 54%. Prior to placing, all of the layers were binded using the binder and heat gun was used to activate the binder.

**Figure 2:**
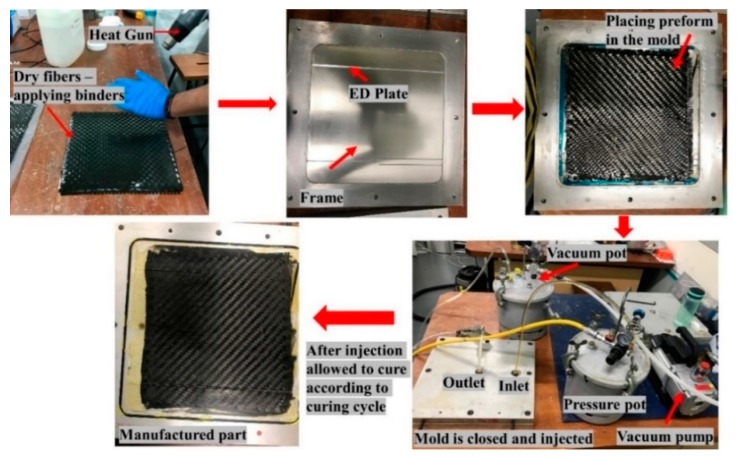
Manufacturing steps during the Resin Transfer Moulding (RTM) of composite laminate.

The top mold was closed after the preform of dry fibres was placed along with the frame. Once the mould was closed, the Elium^®^ resin was prepared at a weight ratio of 100:3 with benzoyl peroxide and it was injected at room temperature (RT) at 2-bar injection pressure. As shown in Figure 1b, the resin flows circumferentially in the mold and fills into the dry fibers. After the fibers are completely injected, the excess resin flows out through the outlet. Once the excess resin comes at the outlet, the inlet and outlet were both closed, and the laminate was allowed to cure at RT. Later, once the laminate is cured, it was post-cured at 65 °C on the hot plate for 45 min. before demoulding. Figure 2 shows the general steps for the manufacturing of the composite laminate using the RTM manufacturing technique. 

### 2.3. Manufacturing of ED Integrated Thermoplastic Composite Laminates 

For welding study, thermoplastic composite laminates were manufactured with ED of varied configurations, namely semi-circular, triangular, and flat, as recommended by many researchers in the literature [25,34,64]. The below subsections will give further details regarding the entire manufacturing process.

#### 2.3.1. Mold Design

**Figure 3:**
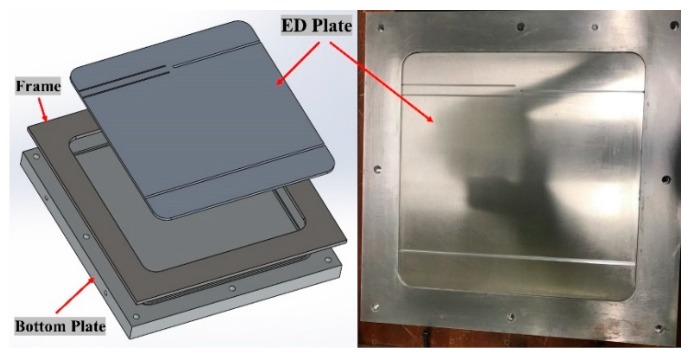
Mold design of Energy director (ED) composite laminate manufacturing.

Four-part (top mold, bottom mold, frame, and ED plate) mold was conceptualized to be used for the manufacturing of ED integrated composite laminates (refer Figure 3). Two configurations of semi-circular energy directors were manufactured, namely single semi-circular Elium^®^ composite laminate (SC-ELC) and double semi-circular Elium^®^ composite laminate (2SC-ELC), as described in Table 2. 

ED plates of 4 mm thickness with different sizes of grooves, according to the desired volume and the placement strategies were manufactured (refer Figure 3). A 6 mm frame was used, wherein a 4 mm ED mold plate was inserted to give the final thickness of 2 mm. Figure 3 shows the mould design of the manufacturing of the integrated ED composite laminate.

#### 2.3.2. Manufacturing

For the manufacturing of integrated ED composite laminate, a similar manufacturing process, as used for the flat laminate, was adopted. After placing the dry preform on the ED grooved plates of 4 mm thickness, a frame of 6 mm thickness was placed, and the resin was injected after closing the mould. Once the resin was injected and cured, a pure resin chunk was obtained as an energy director integrated with the laminate due to the ED grove plate. The injection parameters used for integrated ED laminate were obtained by different manufacturing trials. Previous researches have shown a need to optimize the amount of vacuum, as there are chances of increasing the porosity with an uncontrolled vacuum when the monomer reaches the exotherm [5,63]. 

For integrated ED manufacturing trials, initially, manufacturing was tried with six layers of dry carbon fibre to obtain a 2 mm thick panel, which resulted in a fibre volume fraction (V_f_) of 64%. As the welding phenomenon of the thermoplastic composite is resin dominating, the required fibre volume fraction should be lesser than the ideally desired high V_f_ composite parts [34,42]. Subsequently, all of the trials were carried out with five layers of fibers, which resulted in the fibre volume fraction of 54% for a 2 mm thick panel. The laminate was found to have a good surface finish, but the EDs contained some microvoids. Subsequently, the laminate injection pressure was kept at 2-bar pressure, but the outlet was kept at a partial vacuum of 505 mbar with a view to remove the entrapped air in the mould before injection, but the manufactured ED integrated laminate still showed small bubble content. The inlet condition was changed, and the resin was injected at 5-bar pressure keeping the similar outlet condition of 505 mbar vacuum in order to reduce the amount of bubbles in the ED. The manufactured laminate has shown significant fibre movement due to high pressure but the bubbles in the ED were significantly reduced.

A trial was also done by filling the ED initially with resin and then placing the dry fibers on them. After closing the mould, the resin was injected at an inlet pressure of 2-bar and the outlet was kept at a vacuum of 505 mbar. This condition did not show any improvement as well. All of these initial trials were carried out with resin viscosity of 100 cP. Later, few trails were also tried using high viscosity resin of 200 cP and low viscosity resin of 50 cP at the same inlet and outlet conditions. Low viscosity resin (50 cP) was tried with the aim of fastening the injection and reducing the possibility and amount of entrapped air in the grooves. While the higher viscosity resin (200 cP) was used with a view to slow down the resin injection and possibly reduce the air bubble that may be created due to race-tacking during the injection with the lower viscosity. Injected panels with higher viscosity resin showed the undesired movement of the fibers and there was still the air entrapped in the ED. Additionally, no significant improvement was noticed with 50 cP resin. It is to be noted that three trials for each condition were performed to check the repeatability of the result. Table 3 shows a summary of the important manufacturing trials that were carried out in the current project.

**Figure 4:**
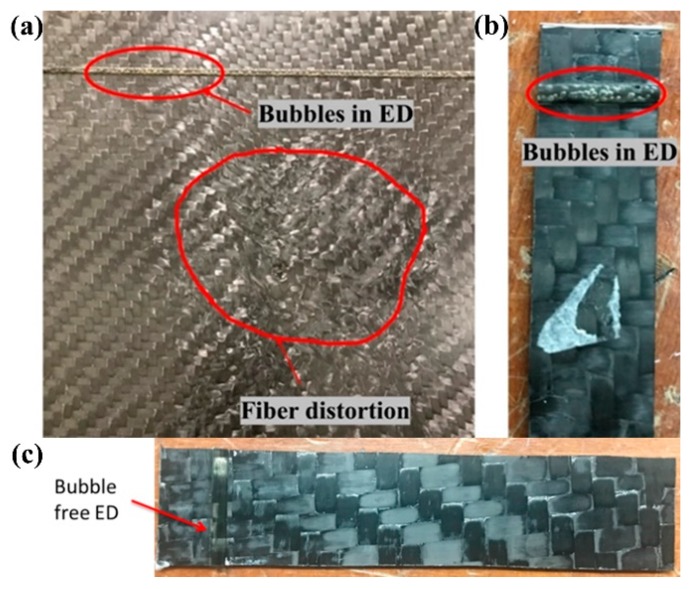
(**a**) Manufacturing trial showing the bubbles and fiber distortion, injected using 200 cP resin; (**b**) Bubbles entrapped in the ED during manufacturing at 100 cP, 2-bar injection pressure and 505 mbar outlet vacuum; and, (**c**) Manufactured bubble-free ED laminate with recommended parameters.

Figure 4 shows the manufactured samples of some of the trials where 4a depicts the entrapped in the ED and fibre movements during the injection with high viscosity 200 cP resin. Figure 4b shows an example of how the bubbles are entrapped in the ED during different manufacturing trial with 100 cP at an injection pressure of 2-bar and outlet at 505 mbar vacuum. After few trials with different combinations, integrated ED laminate with almost bubble-free and negligible entrapped air was obtained. The optimum resin viscosity was found to be 100 cP, with an injection pressure of 2-bar and 270 mbar vacuum at the outlet. Resin degassing should be carried at 65-mbar for 10 min. At the start of the injection, mould was kept at a full vacuum to evacuate the entrapped air and the resin was injected at 1.2-bar pressure. Immediately after the injection starts, inlet pressure is increased to 2-bar and outlet vacuum is reduced to 270 mbar and the panel was injected at this pressure condition.

**Figure 5:**
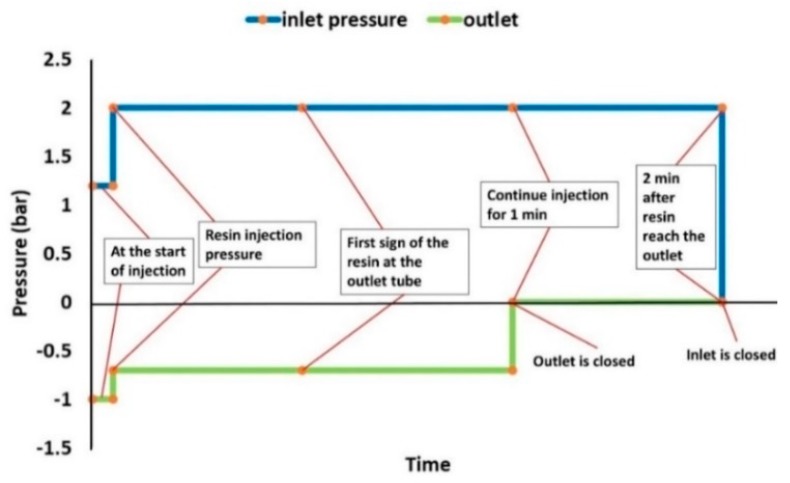
Resin Transfer Moulding (RTM) injection strategy for ED integrated panels.

Figure 5 illustrates the recommended injection scheme for the manufacturing of integrated ED laminates. 

All of the manufactured laminates have undergone void content measurements. ASTM D792 [65] and ASTM D2734-09 [66] were used to quantify the void content in the composite laminates and the laminates with void content <1% were used for welding trials.

Laminate with a triangular ED configuration was manufactured with the same procedure mentioned in the manufacturing of semi-circular ED. Few trials were carried out for triangular ED manufacturing, but the bubble content was much higher than the semi-circular ED. Significant manufacturing trials are required to achieve a bubble-free ED integrated laminate. Additionally, from the literature, it was noticed that both triangular and semi-circular have shown comparable lap shear performance [7,38]. Hence, it was decided to further the research by finding the recommended injection parameters for only SC-ELC integrated panels.

For the manufacturing of Elium^®^ film, a two-part mould with a frame of different thickness of 0.25 mm and 0.5 mm were used and manufactured while using the RTM process with the injection of neat Elium^®^ resin into the mould.

## 3. Experiments 

### 3.1. Welding Methodology

**Figure 6:**
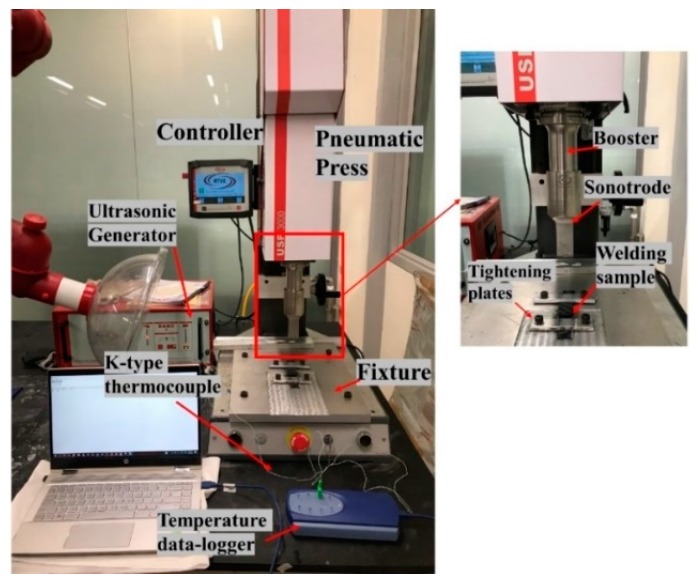
Details of the ultrasonic welding equipment used in the current project.

Ultrasonic welding machine with a generator of 20 kHz frequency, a maximum power output of 3000W, and AWC-6 microprocessor controller was used for the welding of specimens in the current project, as shown in Figure 6. In current research work, a constant time mode of welding was used for weld quality investigation. In the constant time mode, four parameters are required for welding, namely weld time, hold time, weld pressure, and the amplitude. In the current research, four parameters (weld time, weld pressure, amplitude, and energy directors) will be investigated for the optimization of the welding process. These parameters are later explained in detail in Section 4.1, along with the initial trials and welding optimisation study. 

The max amplitude (i.e., at 100%) that is available at the tip of the sonotrode is 65 µm for this welding machine. Standard (ASTM D5868-01) lap-shear specimens were manufactured to carry out the welding study. A fixture was designed to weld the lap joint samples and, for that, a step of 2 mm was milled to balance the offset with the specimen thickness of 2 mm (Refer Figure 6). After placing the specimen into the fixture, it was fixed by the tightening mechanism to ensure that the sample does not move during the welding process. 

**Figure 7:**
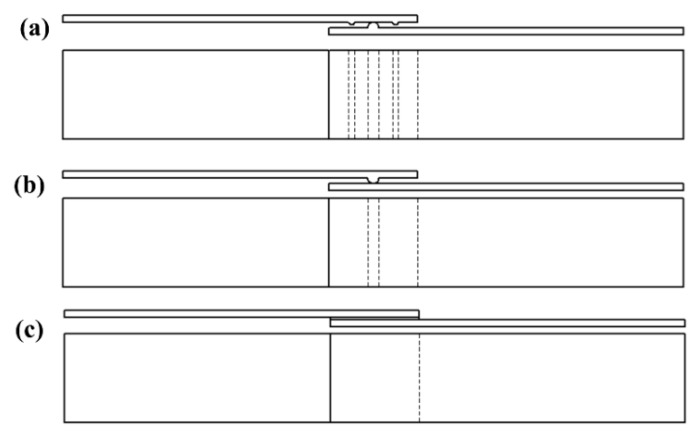
Schematic of welding configurations used (**a**) SC-ELC_2SC-ELC; (**b**) SC-ELC_FL-ELC; and, (**c**) FL-ELC_FL-ELC (ELF 0.5 mm and 0.25 mm).

Different types of energy directors used in the current research work were semi-circular ED composite laminate (SC-ELC), Double semi-circular ED composite laminate (2SC-ELC), and Flat ED of 0.25 and 0.5 mm thickness. The different welding configurations that are investigated and optimized are SC-ELC_2SC-ELC, SC-ELC_FL-ELC, and FL-ELC_FL-ELC (Elium^®^ Film (ELF)/flat ED of 0.5 mm and 0.25 mm thickness). Figure 7 details the schematic of the following welding configurations.

### 3.2. Lap Shear Test

Lap shear test is widely performed to evaluate the joint strength due to its ease of use as well as excellent reproducibility in the results [67]. The lap shear test was performed according to the standard ASTM D5868-01 [68] with a specimen of dimensions, length = 101.6 ± 0.2 mm, width = 25.4 ± 0.1 mm, thickness = 2 ± 0.01 mm, and overlap area = 25.4 × 25.4 mm^2^. A universal testing machine INSTRON of 50 kN load capacity was used for performing this test. The test was performed with a crosshead speed of 13 mm/min. and was carried out at room temperature/ambient conditions. The load-displacement curve was given by the Bluehill 4 interface software, which was attached to the machine.

Two different lap shear strength (LSS) was calculated, as suggested by Villegas, I.F. et al. [25] due to a difference in the welded area, LSS1, and LSS2. LSS1 was calculated as the peak load divided by the total overlap area and LSS2 was calculated as the peak load divided by the effective welded area. Thus, LSS1 defines the effectiveness of the joint and LSS2 defines the weld quality. The effective welded area was calculated by observing the fractured surface of the tested specimen. ImageJ software (1.8.0_112) was used to calculate the effective welded area by calibrating the length and using a free tool to mark and calculate the area. Three trials were performed for each welding condition.

## 4. Results and Discussion

### 4.1. Initial Experimental Trials

The maximum amplitude in the current welding machine is 65 µm i.e., at 100%. Initially, For integrated ED, all of the trials were carried out at 100% amplitude with varying weld time and weld pressure. However, the welded samples resulted in (1) the excess amount of resin flowing out of the interface and (2) massive delamination of the top adherend. At 100% amplitude, the samples were also welded at the low weld time and low pressure, but the same phenomenon was observed. This phenomenon is attributed to the higher energy transfer at the weld interface. So, the amplitude was further reduced, and the samples were welded at 75% (48.75 µm). The welded samples showed more promising results in lap shear tests when compared to the one with 100% weld condition. Additionally, from the literature, it is advised to weld the acrylic-based matrix in the range of 40–70 µm amplitude [69] and this amplitude falls within the desirable limit. Few samples were welded at 50% amplitude, but the energy director was not melted and there was a visible gap between the adherends. In most of the research available [34,35], the amplitude was kept constant and other parameters were varied, so in the current investigation, for integrated ED samples, 75% (48.75 µm) amplitude was used as a fixed parameter in the welding optimization study. 

Similarly, for weld time for integrated ED, the samples were initially welded from 0.5 s to 3 s. The results showed that, when samples were welded with higher welding time i.e., above 2 s, there is significant damage to the sample. At higher weld time, the top adherend was damaged by delamination and fibre crushing. The adherend failed by a minimal manual force as it is over-bonded and damaged. Thus, the range of welding for the current project was selected to be from 0.5 s to 2 s with 0.5 s interval. Initially, the weld pressure used was from 1-bar to 6-bar. Weld pressure that was higher than the 5-bar showed a similar phenomenon of the delamination of the upper adherend while at the pressure lower than 3-bar, the bond was very weak that it can be fractured with manual force. Additionally, visually, a clear gap between the adherends was noticed for an un-bonded joint. In the ultrasonic welding, the energy transferred to the interface is from the top adherend; hence, in the current study for SC-ELC_FL-ELC configuration, SC-ELC adherend was kept on the top. With this methodology, energy directors can be more efficiently used. In SC-ELC_2SC-ELC configuration, SC-ELC adherent was placed at the top. Considering hold time is not the significant weld parameter [35,42], as compared to the other welding parameters, in the current investigation, it was kept constant at 2 s.

For flat laminates with ELF configurations, trials were also carried out at 50%, 75%, and 100% amplitude. For flat laminates, at 100%, and even at 75%, the top adherend showed the delamination and fiber breakage. But at 50% amplitude (32.5 µm) it showed more promising results. Similarly, for the flat welding configuration, initial trials were carried out with the same time range of the above-mentioned ED integrated configurations, but the bonding was found to be excessively too weak and it broke by manual force. Further adherends were tried to be welded from 3.5 s to 6 s. At 3.5 s, the bond was still weak, but it showed better results from 4 s onwards. But at the higher weld time of 6 s, adherends were found to be over-bonded. Accordingly, in the current study, a weld time of 4 s to 5.5 s was selected with a 0.5 s interval. The weld pressure was kept the same for flat ED configuration as with the above-mentioned ED integrated configurations i.e., 3-, 4-, and 5-bar. Initial trials were also carried out to understand the film thickness effect with 0.25, 0.5, and 0.9 mm ELF. For 0.9 mm thick film, proper bonding was not obtained between the adherends due to the presence of an excess amount of the resin, which does not help in welding. Whereas, for 0.25 mm and 0.5 mm ELF showed better results. Hence, in the current study detailed optimization is carried out with 0.25 and 0.5 mm thick ELF. For all of the configurations with flat laminates, the hold time was kept constant at 4 s.

**Figure 8:**
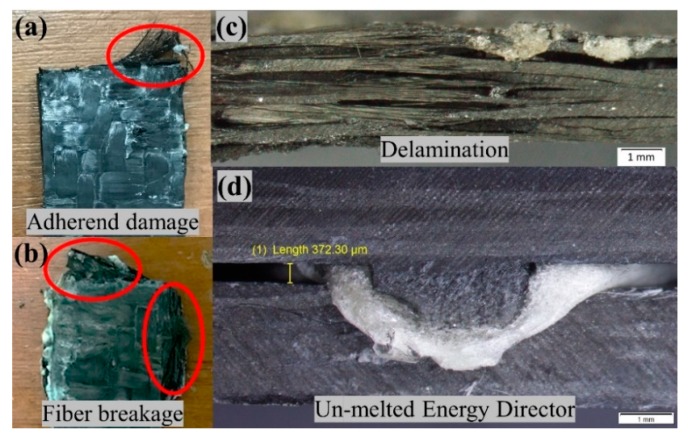
Initial welding trials (**a**,**b**) over-bonded when welded at higher pressure of 5-bar (**c**) delamination observed when welded at higher amplitude of 100% (**d**) un-bonded joints when welded at a lower pressure of 2-bar.

Figure 8 shows the over bonded (a–c) and under-bonded (d) welded specimen of the initial trials. Where clear delamination and the fiber breakage can be observed in the over-bonded specimen, while an un-melted ED can be observed for under bonded specimens.

**Figure 9:**
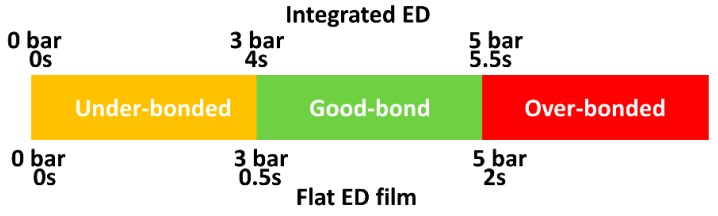
Effect of welding parameters on bonding for integrated and flat ED composites.

Figure 9 shows a summary of the initial trials and welding parameters (weld time and weld pressure) effect on the bonding conditions. 

In the current research, the full factorial design (FFD) was used as the design of experiments. From the initial trails, as mentioned above, the following are the welding parameters selected for the welding of the adherends with the different weld configurations shown in Table 4.

Analysis of variance (ANOVA) is a statistical method that is used to find out the impact of the independent factors on the dependent factor in regression analysis and to show the dominating factor which affects the response [70,71]. It checks the impact of one or more factors by comparing the means of different samples. In the current project, ANOVA analysis is used to determine the most significant and influencing factor that affects the lap shear strength (LSS) results. Minitab 19 was used to execute the ANOVA analysis.

### 4.2. Welding of Elium^®^ Composites with Integrated Eds

**Figure 10:**
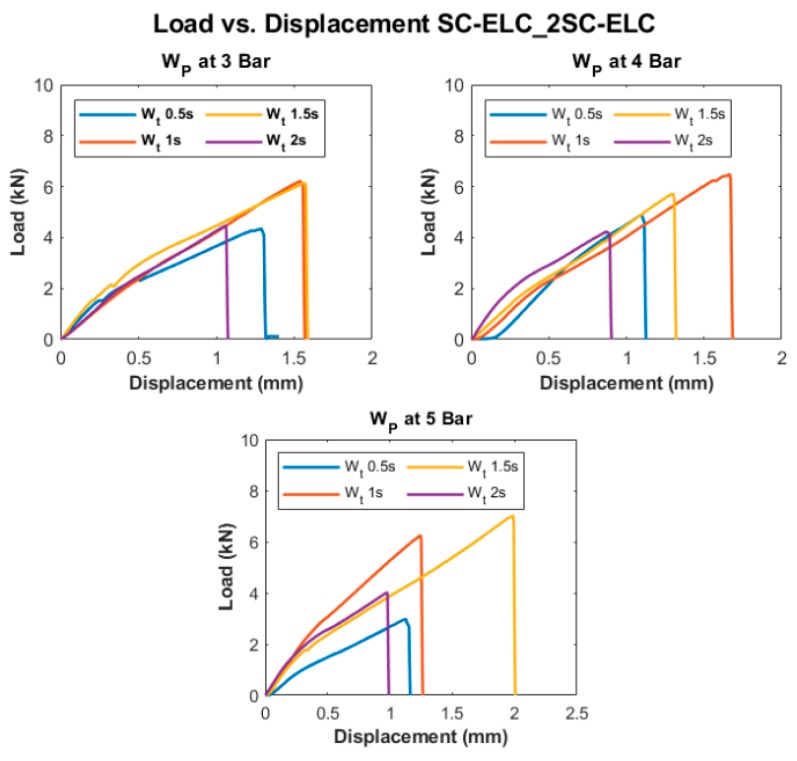
Load vs. Displacement curves of SC-ELC_2SC-ELC configuration at different welding conditions.

**Figure 11:**
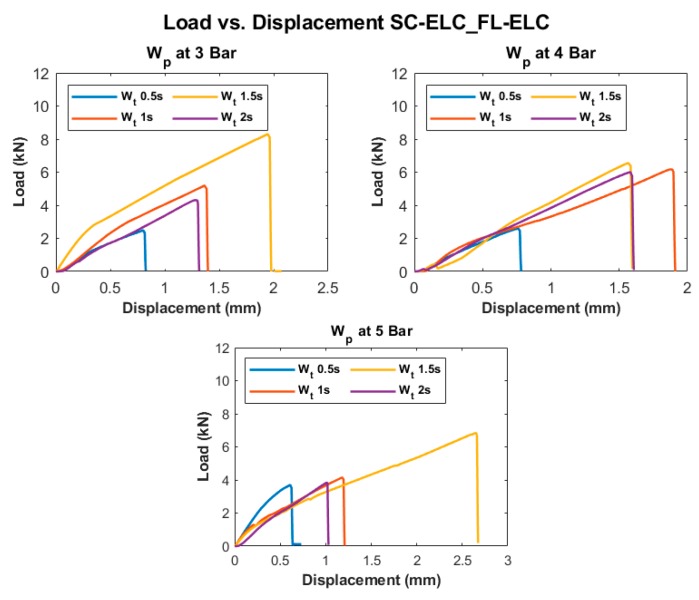
Load vs. Displacement curves of SC-ELC_FL-ELC configuration at different welding conditions.

Figure 10 and Figure 11 show the Load vs. Displacement curves for the welding conditions at three different weld pressure (W_p_) that correspond to four different weld time (W_t_), namely SC-ELC_2SC-ELC and SC-ELC_FL-ELC configuration, respectively. It should be noted that the presented graphs are the best representative curve of the average values of the three trials at each welding condition and the standard deviation of the value is represented in Figure 13 and Figure 14, showing the lap shear strength. A small non-linearity in load vs. displacement curves at the start can be observed, which is usually attributed to the backlash in the testing machine and fixture, being is around 0.1 mm of displacement. Later, the graphs show the linear behaviour until it reached the maximum load, followed by drastic load drop showing the complete failure of the welded bond of the laminate.

Both of the ED integrated configurations, at some constant pressure, the maximum load value keeps increasing with an increase in the weld time up to an instance after which a further increase in weld time shows the reduction of the peak load value or the corresponding weld strength of the sample, as depicted Figure 10 and Figure 11. The weld time after which the load starts reducing represents the maximum/optimal weld strength condition corresponding to a specific pressure condition. A further increase in weld time after the optimal scenario will keep reducing the weld strength. Tao, W. et al. observed a similar effect, where they have investigated the ultrasonic welding of CF/PEEK composites at a different weld time with and without ED [35]. Similarly, it can also be depicted from the graphs that the displacement representing the elongation also follows a similar trend; it increases to a maximum value corresponding to the maximum load value and then it starts reducing. 

Displacement and weld strength changes can both be explained, as, with the increase in weld time, the amount of weld energy at the interface increases as at a specific pressure, the weld time is directly proportional to the weld energy that is transferred to the adherend [72]. Higher energy at the interface helps in the melting and flowability characteristics of the energy directors and the adherends can be more efficiently welded and result in increases of the weld strength. Higher energy also helps the frequency to be transferred to the bottom adherend and the matrix to melt and significantly adds to the strength. Once an optimal weld strength has been reached, any further increase in weld energy i.e., weld time, at the interface will reverse the phenomenon and, as a result, there will be excessive melting of resin, which will lead to squeezing it out from the interface. The insufficient amount of resin led to less deformation and the sample easily fails without taking a significant load. On the contrary, at a lower weld time, the amount of energy that is transferred to the interface is not enough to melt the resin and fusion bond it to the bottom adherend. For instance, for SC-ELC configuration at 3-bar pressure, the obtained energy values at 0.5 s, 1 s, 1.5 s, and 2 s were 456.65 J, 903.55 J, 1243.1 J, and 1598.5 J, respectively. The energy values clearly show the trend of increase with the increase in weld time at a particular weld pressure. At 0.5 s weld time, the energy of 456.65 J is too low for the welding and, hence, resulted in lower weld strength. On the contrary, at weld time of 2 s, the amount of energy i.e., 1598.5 J was found to be significantly higher for the welding and a drastic drop of peak load can be observed. A similar phenomenon of energy was observed for all of the weld conditions.

The load values from the Load vs Displacement graph, as discussed in the previous section, is used to identify the weld effectiveness and weld quality. Two different LSS values were calculated, LSS1 and LSS2, respectively, for both the configuration of welding. LSS1 was calculated while using the total overlap area (25.4 × 25.4 mm^2^) and LSS2 was calculated using the actual welded area of the samples. 

**Figure 12:**
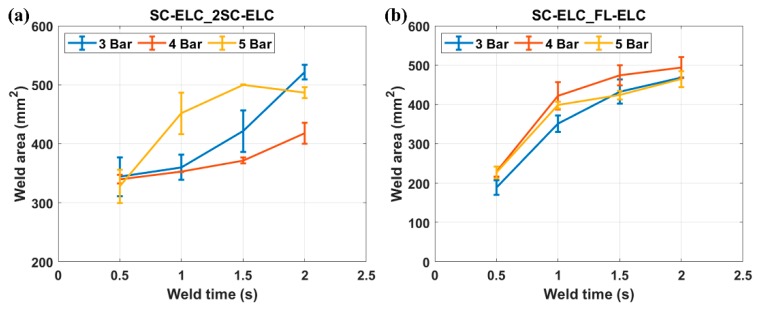
Welded area under all the welding conditions for (**a**) SC-ELC_2SC-ELC (**b**) SC-ELC_FL-ELC.

The actual welded area was calculated for all of the welding conditions using *ImageJ* software for SC-ELC_2SC-ELC and SC-ELC_FL-ELC configurations, as shown in Figure 12. Figure 12 clearly shows that the welded area increases with the increase in weld time for each weld pressure condition. This can be explained as with the increase in weld time, the energy concentration at the interface increases, and in turn elevates the temperature at the interface. This resulted in more melting of resin and it flows over a larger area.

**Figure 13:**
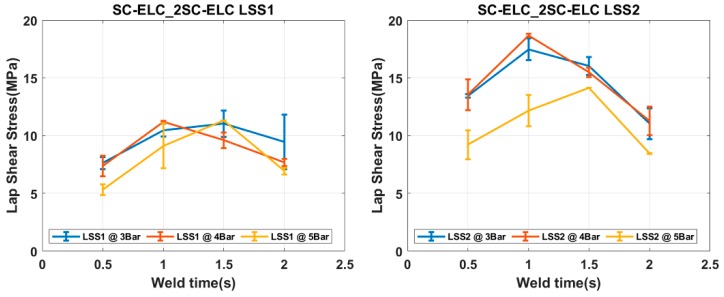
LSS1 and LSS2 graphs for SC-ELC_2SC-ELC configuration.

**Figure 14:**
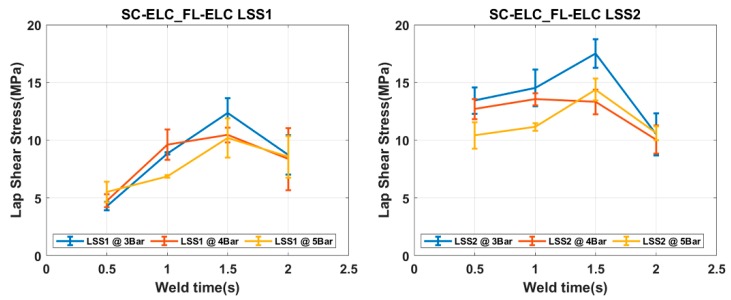
LSS1 and LSS2 graphs for SC-ELC_FL-ELC configuration.

Figure 13 and Figure 14 depict the LSS1 and LSS2 values of the specimens at different welding conditions for SC-ELC_2SC-ELC and SC-ELC_FL-ELC configurations, respectively. The lap shear curves show a similar trend as can be observed in the load-displacement curve. Figure 13 and Figure 14 show that the LSS value increases with an increase in the weld time at specific pressure the but after the optimal weld time has reached, it starts reducing. Further increasing weld time significantly reduces the LSS value. This is due to more energy concentration during welding for a longer period. At higher energy, the temperature at the interface also rises, which led to fiber breakage, delamination, and other phenomenon. These phenomena were dominant in both configurations and reduced the weld strength. It will be explained in more detail later in the section. In both the configurations, LSS2 graphs show that with the increase in the weld pressure, the weld strength remains constant or there is a slight reduction. The results at 3-bar and 4-bar has shown nearly similar weld strength, but for both the configuration at 5-bar, it has shown a drastic reduction in the weld strength. It can be explained as, at higher pressure, the resin squeezes out of the interface and it also damages the adherend, which reduces the weld strength. 

From Figure 13, it can be observed that, for SC-ELC_2SC-ELC configuration, the maximum LSS2 value of 18.68 MPa was obtained at weld time of 1 s and 4-bar pressure, and minimum LSS2 value 8.45 MPa was found at 2 s weld time and 5-bar weld pressure.

**Figure 15:**
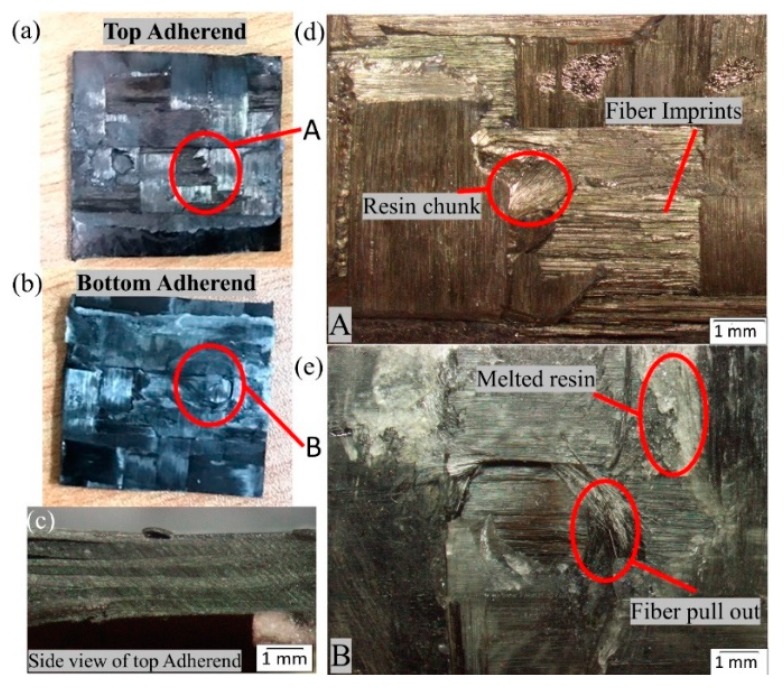
Fracture surfaces of SC-ELC_2SC-ELC specimen with maximum lap shear strength (LSS) value.

**Figure 16:**
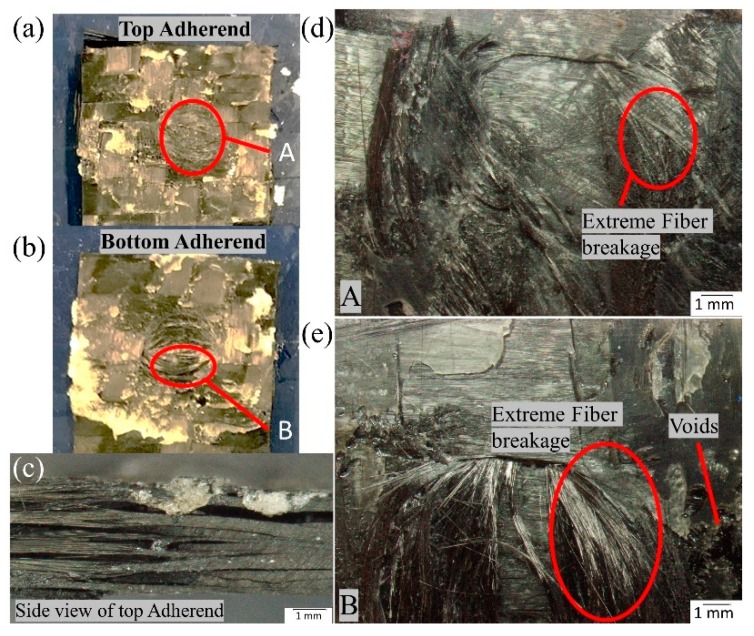
Fracture surfaces of SC-ELC_2SC-ELC specimen with minimum LSS value.

A microscopic investigation was also carried out to understand the failure mechanisms and modes of failure. In the current study, the fracture surfaces of the specimen with the highest and the lowest lap shear strength values were analysed. Figure 15 and Figure 16 show the microscopic image of the top and bottom adherend of the maximum lap shear strength value and minimum lap shear strength, respectively, for SC-ELC_2SC-ELC configuration.

Figure 15a and Figure 16a show the fractured surfaces of top adherend, while Figure 15b and Figure 16b show the fractured surfaces of bottom adherend and Figure 15c and Figure 16c show the top view of the top adherends with maximum and minimum lap shear strength, respectively. As seen from Figure 15, at point A, fiber impingement can be observed along with fiber imprints and the melted resin chunk. At point B, the fiber pulls out and also the melted resin can be observed. These observations are evident in good interfacial bonding and they represent the cohesive failure of the specimen. Whereas, in Figure 16 at a point, A it can be seen that there is a bundle of fiber is pulled out, and there is fiber breakage and also in bottom adherend at point B clear fiber breakage and voids can be seen, which represent the higher energy accumulation at the interface due to higher weld time and pressure condition, resulting in the poor adhesion between the adherend. This can also be observed by comparing Figure 15c and Figure 16c, which shows the amount of delamination that occurred at the adherend. Additionally, for the sample with minimum LSS value, the adherend is found to be delaminated more and, hence, it cannot play part in providing the strength, as it drastically fails during the static lap shear test. From Figure 13, at 5-bar pressure, it was noticed that when the weld time is increased from 1.5 s to 2 s, the LSS2 value drops from 14.13 MPa to 8.45 MPa. Similar results can be seen for all of the pressure conditions with corresponding higher weld time parameters.

### 4.3. Welding of Elium^®^ Composites with Flat EDs

Pure Elium^®^ films (ELF) were used for the welding of flat Elium^®^ composite samples without integrated energy directors. Two different thicknesses of films were analysed 0.5 and 0.25 mm. Twelve different welding conditions were examined with both the film thicknesses configurations, as discussed in the previous Section 4.1. of initial trails.

**Figure 17:**
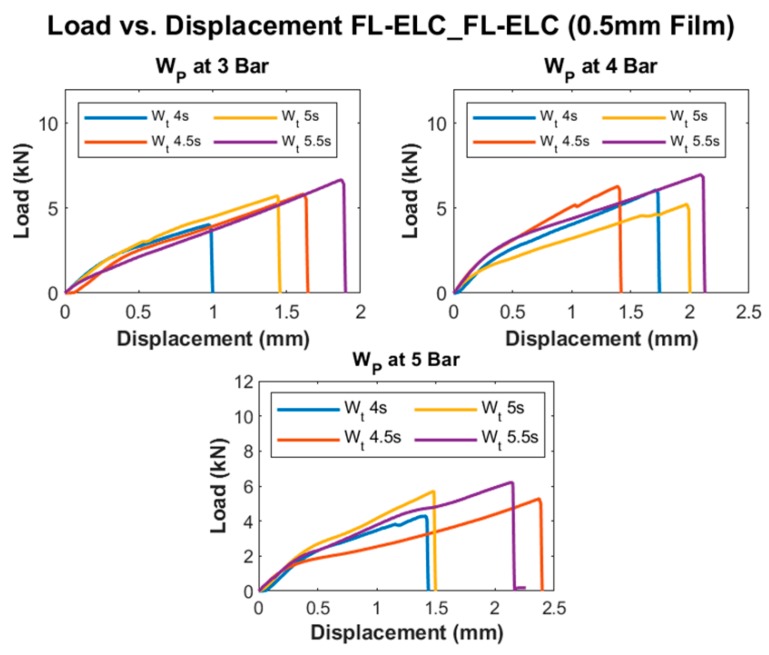
Load vs. Displacement curve of FL-ELC_FL-ELC (0.5 mm film ED).

**Figure 18:**
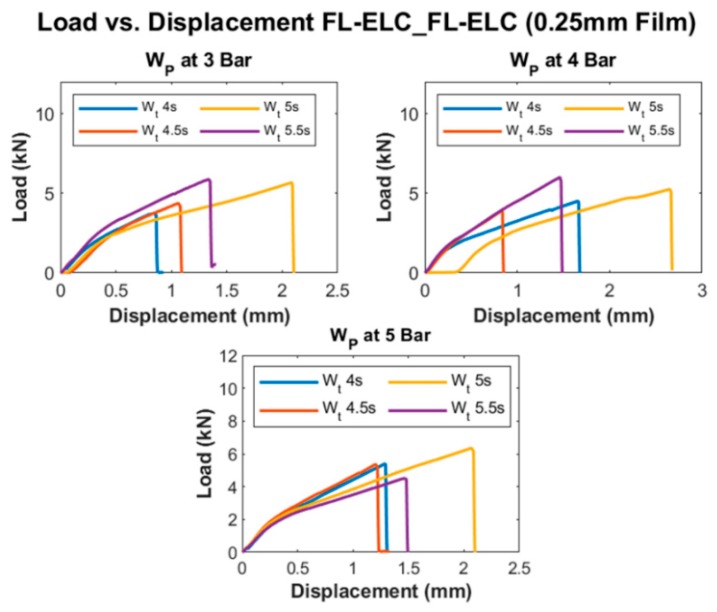
Load vs. Displacement curve of FL-ELC_FL-ELC (0.25 mm film ED).

Figure 17 and Figure 18 show the load vs. displacement graphs for all of the welding conditions of FL-ELC_FL-ELC configuration with 0.5 mm and 0.25 mm thickness ELF, respectively.

**Figure 19:**
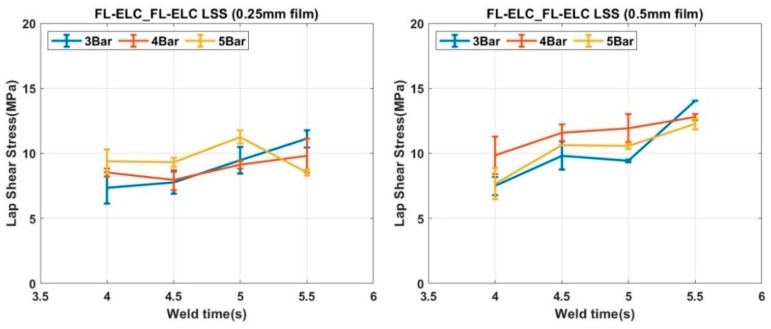
LSS values of FL-ELC_FL-ELC (0.25 mm and 0.5 mm ED film).

Figure 17 and Figure 18 show a similar trend, as discussed previously for integrated ED configuration, where the load value increases with the increase in weld time for the given pressure condition up to a certain limit and further increase in weld time reduces the peak load. For a 0.5 mm film configuration specimen, the peak load was attained at 5.5 s for all three pressure conditions. This is more evident in Figure 19, showing the LSS values at different weld conditions. It was reported from the initial trials that with the increase in weld time to 6 s, top adherend was completely damaged with the fiber crushing as the local heat accumulation at the interface was much higher, which also leads to forming significant voids and deteriorating the weld quality. 

For the welding of FL-ELC configuration, a resin film of the same dimension was used for the welding as of overlap area. Accordingly, the total overlap area and the welded area were the same, so only one LSS value is determined for this configuration. Figure 19 shows the LSS value for all of the welding conditions of welding of FL-ELC configurations with 0.5 mm and 0.25 mm film thickness, respectively. The maximum LSS value for the 0.25 mm film configuration was obtained at the weld pressure of 5-bar and weld time of 5 s, while for 0.5 mm film configuration, it was obtained at weld pressure of 3-bar and weld time of 5.5 s. A thin film of 0.25 mm thickness required higher pressure (5 bar) and higher time (5 s) to be fused with the bottom adherend to obtain the maximum LSS, while 0.5 mm film can be welded at lower weld pressure (3 bar) and 5.5 s, as seen from Figure 19. The usage of lower thickness ED film leads to higher cyclic strains and hence results in the increase in heat generation on both the adherends. Hence, below a certain thickness, there is a risk of overheating of the interface due to the simultaneous heating and melting of the ED and the adherend [73]. Hence, in the current investigation, the ED with 0.25 mm thickness yielded lower weld strength as compared to the ED with 0.5 mm thickness.

**Figure 20:**
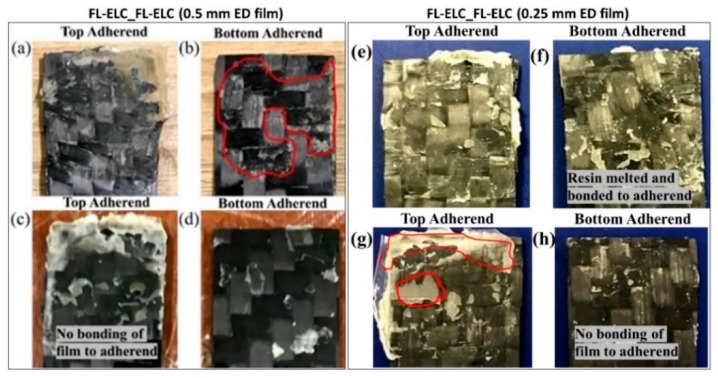
Fracture images of the top and bottom adherends for maximum LSS (**a**,**b**) and minimum LSS (**c**,**d**) FL-ELC_FL-ELC (0.5 mm ED film) and maximum LSS (**e**,**f**) and minimum LSS (**g**,**h**) for FL-ELC_FL-ELC (0.25 mm ED film).

The fractured samples of the FL-ELC configuration did not show much fiber impingement or fiber damage as observed in the SC-ELC_2SC-ELC and SC-ELC_FL-ELC configurations due to lower viscoelastic heating in the flat film ED as compared to the semi-circular ED. Therefore, for the FL-ELC configuration, visual observations were performed and found to be sufficient for understanding the failure mechanism. Figure 20 shows the failure images of the top and bottom adherend of the maximum LSS (a,b) and minimum LSS (c,d) under lap shear testing for FL-ELC_FL-ELC configuration with 0.5 mm ELF. The minimum LSS value for 0.5 mm ELF configuration was found at the weld time of 4 s and weld pressure of 3-bar, whereas the maximum LSS value was achieved at weld time of 5.5 s and weld pressure of 3-bar. 

From Figure 20a, the melting of the resin film and its integration with the adherend can be seen, whereas, in Figure 20c, a clear film can be seen at the top adherend, which is not melted, represents the insufficient energy at the interface for bonding. While comparing Figure 20b,d, for the laminate with maximum LSS condition, it was noticed that the resin at the bottom adherend marked was partially bonded with the adherend showing that the failure more is partial cohesive model, on the other hand, in Figure 20d it is evident that the failure mode is purely adhesive failure owing to the lesser weld time of 4 s and weld pressure of 3-bar.

Figure 20e–h shows the fractured images of the FL-ELC adherends welded with 0.25 mm Elium^®^ film at the interface between the adherends. Figure 20e,f shows the adherends with maximum LSS value, whereas Figure 20e,f depicts the one with minimum LSS value. From Figure 20e,f, it can be seen that there is a melting of the resin and melted resin can be found on both the top and bottom adherends whereas, from Figure 20g,h, an adhesive failure can be seen i.e., film has not formed a good bond with the bottom adherend. Figure 20g shows an un-melted resin chunk, which suggests that there was insufficient energy at the interface to melt the Elium^®^ film, hence it formed a weaker bond. 

As for the minimum LSS weld condition, there was lesser weld time and the weld pressure of 4 s and 3-bar respectively as opposed to maximum LSS welding condition with weld time and weld pressure are 5 s and 5-bar, respectively. For the FL-ELC_FL-ELC configuration, the LSS value for different film thicknesses was also compared. It was noticed that the welding of flat samples with 0.5 mm film gives around 25% higher LSS when compared with the flat samples that were welded with 0.25 mm Elium^®^ film. As the film thickness reduces, weld pressure required to form a good bond also increases i.e., 0.25 mm film welding adherend required 5-bar welding pressure, whereas the samples that were welded with 0.5 mm film only required 3-bar of pressure to obtain an optimal bonding between the adherends. 

Few trials were also carried out to check the weld results of flat samples while using significantly higher volume resin film i.e., Elium^®^ film of thickness 0.9 mm, but no improvement in the results was obtained. The maximum LSS value that was obtained in the initial trials was 9 MPa, which is significantly lower when compared to the welded sample with 0.5 mm and 0.25 mm Elium^®^ film. An explanation to this can be deduced from the failure mode of the adherend, which was found to be an adhesive failure mode and there was also evidence of extensive squeeze out of the molten resin. This indicates the amount of resin is excessively more than required to form an optimal bond. Similar results were explained by Villegas, I.F. et al. [25] while comparing the different configurations of ED. 

### 4.4. Comparison of Lap Shear Bonding Strength

**Figure 21:**
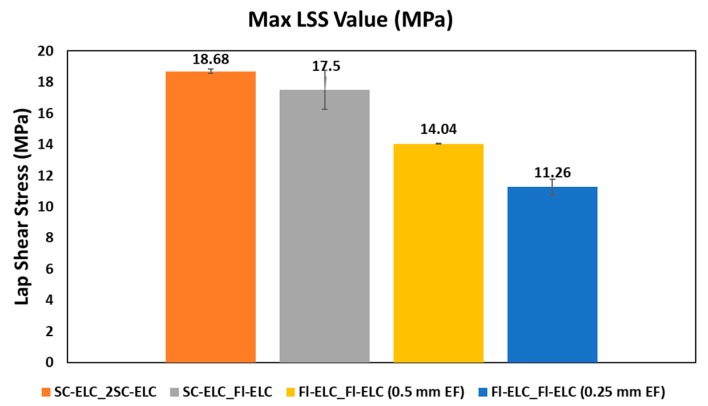
Comparison of LSS for the laminates welded with different ED configurations.

Figure 21 shows the comparison of the LSS values of all the welding configurations that were performed in the current research. It is to be noted that the comparison for welding is done using LSS2 value, which is the measure of the weld quality. It can be clearly seen that, among all the welding configurations, SC-ELC_2SC-ELC shows the best result with 18.68 MPa, as seen from Figure 21. Between SC-ELC_2SC-ELC and SC-ELC_FL-ELC configurations, there is not much to separate in terms of LSS values. FL-ELC_FL-ELC (0.5 mm ELF) shows a 35% lower LSS value when compared to ED integrated SC-ELC_2SC-ELC samples showing the effectiveness of the energy director in the ultrasonic welding. From the failure mechanism study, it was noticed that the energy directors helped to concentrate more energy at the interface and, hence, resulted in higher LSS value when compared to the Flat ED configuration. The weld time for the maximum LSS2 value for SC-ELC_2SC-ELC configuration was 1s, while that of FL-ELC_FL_ELC (0.5 mm ELF) is 5.5s. Semi-circular energy directors are found not only to increase the weld strength, but it is also up to four times faster the welding, which is carried out with a 0.5 mm flat ED film. 

### 4.5. Fractography

**Figure 22:**
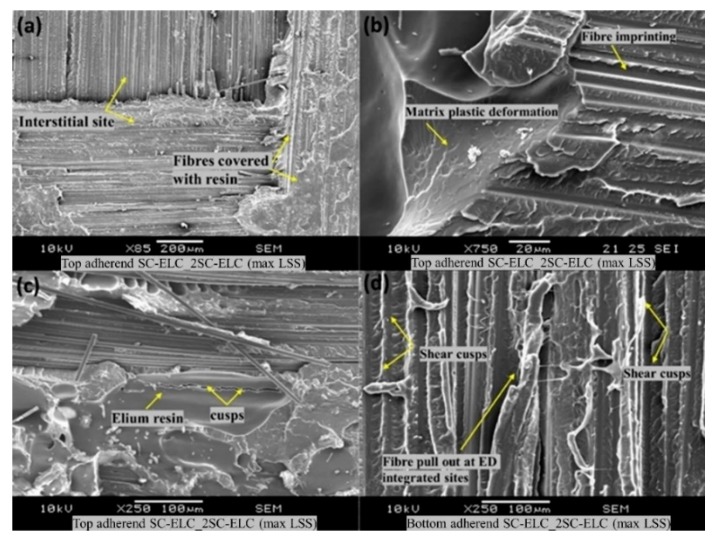
SEM fractography of 2SC-ELC_SC-ELC for maximum LSS (**a–c**) represents the failure image of the top adherends showing fibre imprinting, shear cusps and matrix plastic deformation (**d**) represents the failure image of the bottom adherend showing shear cusps and fibre pull at ED sites.

In the present SEM investigation, the fracture surfaces of the SC-ELC_2SC-ELC and SC-ELC_FL-ELC configurations of lap-shear specimens are investigated in detail. The fracture study is analysed based on the lowest and the highest values of the lap-shear samples under investigation. The range of the lap shear strength values for the SC-ELC_2SC-ELC and SC-ELC_FL-ELC configurations was (8.45 ± 0.05 MPa and 18.86 ±0.14 MPa) and (8.42 ±1.14 MPa 1−7.5 ±1.24 MPa), respectively. Hence, the specimens that are examined under scanning electron microscopy are divided into the above-mentioned groups, the sample with minimum and maximum shear strength values for both the ED integrated configurations. Figure 22 shows the surface morphology of the SC-ELC_2SC-ELC composite laminate with the highest shear strength being achieved at welding parameters: 1 s, 4-bar and 75% amplitude (48 µm). During the welding, both the top (with single ED) and the bottom (with two EDs) adherends have different welding phenomenon and they are investigated.

Figure 22a shows the interstitial site of the twill weave reinforcement, where the fibers are covered with the melted resin. Additonally, there is clear evidence of the formation of the plastically deformed zone at the interstitial site, as seen from Figure 22a, and it has been further investigated at higher magnification (refer Figure 22b). As explained in the previous researches [48,74,75], the fracture surfaces noticed in Figure 22a,b is a typical characteristic of slow and ductile fracture, which is attributed to the viscoelastic property of the thermoplastic matrix, herein is the case with Elium^®^ resin. This shows that the flat ED affects the LSS value more than the integrated ED, and the thickness of ED film is an important selection parameter for ultrasonic welding.

Figure 22b also shows the fibre imprinting features on the resin, which was caused due to the excellent carbon fibre impingement/anchorage during the welding process [76]. This feature signifies the excellent carbon fibre/Elium^®^ interfacial bond after welding. Figure 22c shows the formation of the cusps, which results from the application of the load on the fibre bundles during the lap shear testing [77]. This load causes the interlaminar shear at the fiber-matrix interface. The larger shear cusps are evident at the resin dominated sites (refer to Figure 22c), while the smaller shear cusps are found at the vicinity of the fiber-matrix interfaces, where a relatively smaller amount of resin is present (refer to Figure 22d). Similar features were noticed by Barbosa, L.C.M. et al. where the welding performance of PPS/glass fiber composite was investigated [76]. On the bottom adherend, as seen from Figure 22d, the fibers are found to be covered with resin and the fibre pull out is observed at the ED integrated site, which potentially increases the lap shear strength of the specimen.

**Figure 23:**
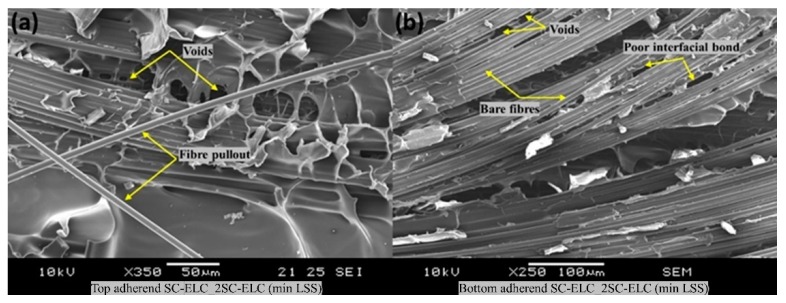
SEM fractography of 2SC-ELC_SC-ELC for minimum LSS (**a**) represents the fractured surface of the top adherend showing voids an fibre pull-out (**b**) represents the fractured surface of the bottom adherend showing voids, bare fibers, and poor interfacial bond.

Figure 23 shows the fractography of the SC-ELC_2SC-ELC composite laminate with the lowest shear strength of 8.45 ±0.05 MPa achieved at welding parameters: 2 s, 5-bar, and 75% amplitude (48 µm). With the increase in the pressure and the weld time, there was an excess amount of resin that was squeezed out, which consequently lead to voids in the welding region due to the presence of insufficient resin (refer Figure 23a,b). Additionally, on both the top and bottom adherends, resin deprived bare fibers are noticed along with the poor interfacial bond. These features significantly lowered the welding efficiency of the SC-ELC_2SC-ELC composite and highlighted the necessity of optimizing the weld parameters.

### 4.6. Analysis of Variance (ANOVA) Results

As discussed in Section 4.1, Analysis of Variance (ANOVA) was performed to statistically evaluate the result of the given welding parameters and find the significant parameter that affects the weld quality by considering LSS as a response function. Separate ANOVA analysis was carried out each configuration, as the welding parameters for integrated ED and flat Elium^®^ film ED configurations were different.

The ANOVA analysis of the integrated ED i.e., SC-ELC_FL-ELC and 2SC-ELC_SC-ELC configurations have four levels for weld time (0.5, 1, 1.5, 2 s), three levels for weld pressure (3-, 4-, and 5- bar), and two ED configurations. Similarly, for flat ED, the ANOVA analysis results have four levels of weld time (4, 4.5, 5, 5.5 s), three levels of weld pressure (3-, 4-, and 5-bar), and two ED film (0.5 mm and 0.25 mm) configuration was carried out. 

The interactions of the above-mentioned parameters and the % contribution of each parameter and their interactions were also carried out and the values are presented in Appendix A for more details. The result of the ANOVA analysis clearly showed that the weld time has the maximum % contribution (53.5%) as compared to the other main factors and their interactions. This shows that the weld time has the maximum influence on the welding and it has a major say in determining the LSS2 value. This can be clearly observed from Figure 13 and Figure 14, where a small change of weld time by 0.5 s resulted in a considerable change in in LSS. Similarly, the results in the literature also state that the weld time is one of the most influencing factors in welding [42,76,78]. ED has the minimum contribution for LSS value for integrated ED configuration. 

For flat ED, ANOVA analysis also showed a similar result and weld time was found to be the most significant factor with a maximum contribution of 40.37%. Whereas, this analysis also confirmed that the weld pressure has a minimum % contribution among the three welding parameters. Interestingly, the ED parameter has also shown a contribution of 19.3%. By increasing the thickness of ELF by 0.25 mm i.e., adherend bonded by 0.5 mm ELF has resulted in an increase in LSS value by 24.6% as compared to adherend bonded by 0.25 mm ELF, as seen from Figure 19. This shows that the flat ED affects the LSS value more than the integrated ED and the thickness of ED film is an important selection parameter for ultrasonic welding.

## 5. Conclusions 

Integrated ED novel carbon/Elium^®^ composites were successfully manufactured and a detailed manufacturing trial study is presented in the current research. An experimental study showing the influence of different welding parameters, such as weld time, weld pressure, amplitude, ED type, and others on the weld strength, were investigated. SC-ELC_2SC-ELC configuration showed the best bonding results with a shear strength of 18.68 MPa. FL-ELC_FL-ELC (0.5 mm ELF) has shown a 35% lower LSS value as compared to ED integrated samples showing the effectiveness of the energy director in ultrasonic welding. The welding results from static lap shear test as well as from ANOVA analysis have shown that the thickness of the ELF affects the lap shear strength (LSS) value more than the different integrated ED and this makes the thickness of ELF an important selection parameter for ultrasonic welding. For all of the ED configurations, lap shear strength value increases with an increase in the weld time at constant pressure, but after the optimal weld time has reached, it starts reducing. Analysis has shown the significant plastic deformation of Elium^®^ resin due to its viscoelastic property and also the shear cusps formation near the resin-rich sites. The cohesive fracture mode was also observed for a specimen at the optimised condition. These observations were typical of the optimised weld condition and they have a direct relationship with strong interfacial bonding. Laminates with lower shear strength have shown voids, poor interfacial bonding, and interfacial failure characteristics.

## Figures and Tables

**Table 1:** Mechanical characteristic of the Elium^®^ 150 resin used in the current project [60,63].

Matrix	Initiator/Hardener	Mixing Ratio by Weight	Density (g/cm^3^)	Viscosity	Shear Strength (MPa)	Glass Transition Temperature (T_g_) °C
Elium^®^ 150	Peroxide	100:3	1.2	100 cP @ 25 °C	22	110

**Table 2:** Energy Director Configurations.

ED Configuration		Size (mm)	Volume (mm^3^)
SC-ELC	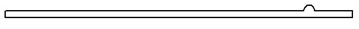	R 1.6 × 25	100.5
2SC-ELC	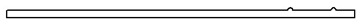	R 0.8 × 25	50.2

**Table 3:** Initial manufacturing trials with different injection parameters for ED integrated composites.

Trial	No. of Layers	V_f_	Inlet Pressure	Outlet Vacuum	Resin Viscosity	Condition	Remarks
1	6	64%	2-bar	505 mbar	100 cP	RTM	Higher V_f_ and bubbles in ED
2	5	54%	2-bar	505 mbar	100 cP	RTM	Bubbles in ED
3	5	54%	5-bar	505 mbar	100 cP	RTM	Fibre distortion and lower bubbles in ED
4	5	54%	2-bar	505 mbar	100 cP	By filling resin in ED before injection- RTM	More bubbles in ED
5	5	54%	2-bar	505 mbar	200 cP	RTM	Fibre distortion and bubbles in ED
6	5	54%	2-bar	505 mbar	50 cP	RTM	Race tracking of resin and bubbles in ED

**Table 4:** Design of experiment for the current project.

Configuration	Weld Time (s)	Weld Pressure (Bar)	Hold Time (s)	Amplitude (%)
SC-ELC_2SC-ELC	0.5, 1, 1.5, 2	3, 4, 5	2	75
SC-ELC_FL-ELC	0.5, 1, 1.5, 2	3, 4, 5	2	75
FL-ELC_FL-ELC	4, 4.5, 5, 5.5	3, 4, 5	4	50

**Figure A1:**
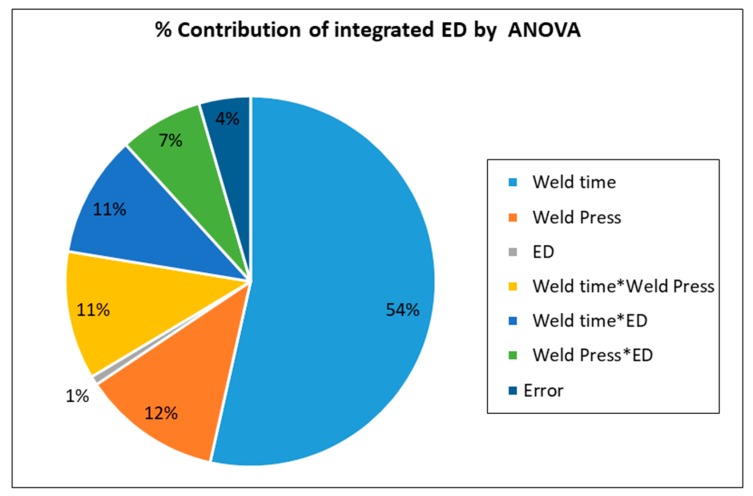
%Contribution of weld parameters for integrated ED film by ANOVA.

**Figure A2:**
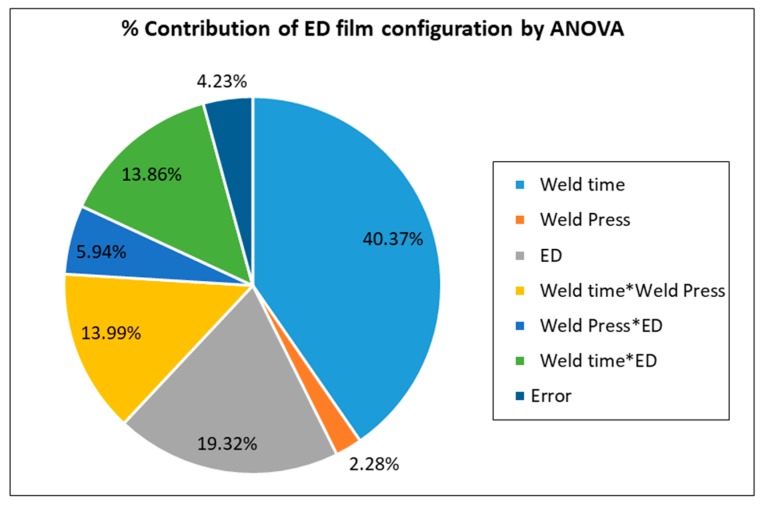
%Contribution of weld parameters for flat ED film by ANOVA.

## Appendix A. ANOVA Results

Table A1 shows the all the calculated parameters for ANOVA analysis of the integrated ED. Figure A1 represents the contribution of the individual parameters for LSS2 value and we can clearly see that weld time is the maximum influencing factor.

**Table A1:** ANOVA analysis for integrated ED (SC-ELC_FL-ELC and 2SC-ELC_SC-ELC).

Analysis of Variance							
Source	DF	Seq SS	Contribution	Adj SS	Adj MS	F-Value	P-Value
**Weld time**	3	97.54	53.51%	97.54	32.51	23.78	0.001
**Weld Press**	2	22.09	12.12%	22.09	11.04	8.08	0.02
**ED**	1	1.435	0.79%	1.435	1.43	1.05	0.345
**Weld time*Weld Press**	6	20.37	11.18%	20.37	3.39	2.48	0.146
**Weld time*ED**	3	19.37	10.63%	19.37	6.45	4.72	0.051
**Weld Press*ED**	2	13.25	7.27%	13.25	6.63	4.85	0.056
**Error**	6	8.20	4.50%	8.204	1.36		
**Total**	23	182.3	100.00%				

Similarly, Table A2 shows the all the calculated parameters for ANOVA analysis of the Flat ED. Figure A2 represents the contribution of the individual parameters for LSS2 value and we can clearly see that weld time is the maximum influencing factor.

**Table A2:** ANOVA analysis for ED film (FL-ELC_FL-ELC (0.5 and 0.25mm film)).

Analysis of Variance							
Source	DF	Seq SS	Contribution	Adj SS	Adj MS	F-Value	P-Value
**Weld Press**	2	1.679	2.28%	1.679	0.8393	1.62	0.274
**Weld time**	3	29.656	40.37%	29.656	9.8854	19.09	0.002
**ED**	1	14.191	19.32%	14.191	14.1911	27.4	0.002
**Weld Press*Weld time**	6	10.279	13.99%	10.279	1.7132	3.31	0.086
**Weld Press*ED**	2	4.367	5.94%	4.367	2.1836	4.22	0.072
**Weld time*ED**	3	10.181	13.86%	10.181	3.3936	6.55	0.025
**Error**	6	3.108	4.23%	3.108	0.518		
**Total**	23	73.461	100.00%				
